# Whatever the Weather: Ambient Temperature Does Not Influence the Proportion of Males Born in New Zealand

**DOI:** 10.1371/journal.pone.0025064

**Published:** 2011-09-21

**Authors:** Barnaby J. Dixson, John Haywood, Philip J. Lester, Diane K. Ormsby

**Affiliations:** 1 School of Biological Sciences, Victoria University of Wellington, Wellington, New Zealand; 2 School of Mathematics, Statistics and Operations Research, Victoria University of Wellington, Wellington, New Zealand; University of Turku, Finland

## Abstract

**Background:**

The proportion of male births has been shown to be over 50% in temperate climates around the world. Given that fluctuations in ambient temperature have previously been shown to affect sex allocation in humans, we examined the hypothesis that ambient temperature predicts fluctuations in the proportion of male births in New Zealand.

**Methodology/Principal Findings:**

We tested three main hypotheses using time series analyses. Firstly, we used historical annual data in New Zealand spanning 1876–2009 to test for a positive effect of ambient temperature on the proportion of male births. The proportion of males born ranged by 3.17%, from 0.504 to 0.520, but no significant relationship was observed between male birth rates and mean annual temperature in the concurrent or previous years. Secondly, we examined whether changes in annual ambient temperature were negatively related to the proportion of male stillbirths from 1929–2009 and whether the proportion of male stillbirths negatively affected the proportion of male live births. We found no evidence that fewer male stillbirths occurred during warmer concurrent or previous years, though a declining trend in the proportion of male stillbirths was observed throughout the data. Thirdly, we tested whether seasonal ambient temperatures, or deviations from those seasonal patterns, were positively related to the proportion of male births using monthly data from 1980–2009. Patterns of male and female births are seasonal, but very similar throughout the year, resulting in a non-seasonal proportion of male births. However, no cross correlations between proportion of male births and lags of temperature were significant.

**Conclusions:**

Results showed, across all hypotheses under examination, that ambient temperatures were not related to the proportion of male births or the proportion of male stillbirths in New Zealand. While there is evidence that temperature may influence human sex allocation elsewhere, such effects of temperature are not universal.

## Introduction

The global secondary sex ratio (SSR: the ratio of male to female births) in humans is currently estimated at 1.07 [1]. This male bias in the SSR deviates from the 1∶1 sex ratio predicted by natural selection [2] and has prompted a large body of research investigating the causal mechanisms underpinning this anomaly. Natural selection may have favoured mechanisms in women that select *in utero* for the offspring that will be most reproductively successful in given environmental circumstances [3]. Political unrest, natural disasters and maternal stress are among a long list of traits suggested to lower the male bias in human sex ratios [4], [5], whereas during the First and Second World Wars in Europe, a more male-biased SSR has been documented [6].

Climatic differences across populations, as well as fluctuations in rainfall and ambient temperature, may also affect the human SSR [7]. Indeed, both increases and decreases in ambient temperature have been shown to have an effect on mortality [8]–[10]. Thus, if climate causes a physiological stress and plays a determining role in sex allocation in humans, it may also affect the SSR if mothers are exposed to shifts in temperature during gestation [11]. According to evolutionary theory [3], there should be fewer males born during stressful periods, as a weaker male would not survive to reproduce where a female might. It has been suggested that more males would be born during warmer periods [12].

The hypothesis that mean annual ambient temperature is related to the SSR has been tested in several studies. Globally the SSR was shown to be significantly less male-biased at tropical latitudes in comparison to temperate and subarctic latitudes [13]. However, studies conducted on a smaller scale in Finland and elsewhere in Scandinavia using long-term data sets and time-series analyses have found that more males are born in years with higher mean annual temperatures [7], [11], [14], which suggests that mean ambient temperature affects maternal condition and influences the SSR. Using annual birth records it is possible to identify patterns in the SSR and potential relationships with environmental factors, such as ambient temperature. However, human conceptions also occur seasonally [15]–[17], prompting the need to investigate any cyclic patterns in SSR using seasonal or monthly data, in order to further identify any adaptive mechanisms of temperature and sex allocation in human beings [18].

Given that the proportion of male births has been shown to be greater in temperate climates around the world [13], and fluctuations in ambient temperature may affect sex allocation in humans [11], [12], [14], we examined whether ambient temperature predicts fluctuations in the proportion of male births in New Zealand. Three separate analyses were conducted, each employing time-series methods. Firstly, we tested the hypothesis that there should be a positive relationship between mean annual ambient temperature and the proportion of males born [7], [11], [14] using historical data spanning 1876–2009. Secondly, we tested the hypothesis that foetal survivability may be influenced by changes in mean annual ambient temperature and may influence the proportion of males born [11], [19] using annual data on the proportion of male stillbirths from 1929–2009. Demographers have noted that despite not having a distinct mating season, human conception rates are greater in the summer months than in the winter months [17]. In a series of studies using data from Germany, Lerchl [16], [18] demonstrated that seasonal patterns in conception rates were also related to the SSR, with more males conceived in summer months, when ambient temperatures were higher. Thus, for our third hypothesis we tested whether seasonal variation in ambient temperature at the time of conception is related to the proportion of male births [18] using monthly data from 1980–2009. We also tested for the effect of extreme temperatures on the proportion of male births using a temperature anomaly series [18] and additionally we considered two simpler hypotheses: that the monthly numbers of births, and the monthly proportions of male births, vary in a seasonal manner [16].

## Materials and Methods

Historically, in New Zealand detailed demographic and vital statistics of European settlers were collected in annual census reports from the mid-1800s by the national statistical office, Statistics New Zealand. From 1925 onwards all ethnic groups were reported, including the indigenous Maori population. Statistics New Zealand provided us with annual numbers of recorded births for the period 1876–2009, which ranged from a minimum of 16,168 in 1876 to a maximum of 65,476 in 1961.

While no demographic dataset will be perfectly accurate, “from the beginning of European settlement New Zealand's demographic data system can be regarded as comprehensive and accurate when compared with those in the most developed countries of the Western World” (p. 10, [20]). However, some demographers have raised concerns regarding the accuracy of birth registrations in New Zealand prior to 1961 [21]. For example, when New Zealand introduced a universal system of family benefits in 1946, birth registrations for Maori were often misreported. In the New Zealand Official Yearbook of 1947–1949 it is stated, “Of the 5776 Maori births registered during 1946 no fewer than 1447 or 25% had occurred before 1945 – i.e. over a year before registration” [21]. Maori birth registrations increased after World War II when legal and financial incentives to register were initiated [22]. However, the definition of Maori ethnicity in census reports has changed over time. Until 1986 census reports used a proportion of blood relatedness. After this, census reports included a self-identification category; however, only individuals who stated solely Maori as their ethnicity were included in ethnic census reports. From 1991 onwards, individuals stating more than one ethnicity were included in ethnic census reports [23]. Due to the different definitions used in census reports we did not separate our analyses by ethnicity. It is important to note that historical registrations of non-Maori births are also subject to measurement error and underreporting prior to 1910 [21]. Late registrations for the total population were also collected post-1910 until the present day, although they were greatly reduced in number compared to previous census reports. Between 1925–1983 late registrations as a proportion of registrations of all live births ranged from 0.2% in 1962 to 1% in 1982, and between 2004–2009 averaged 1.3% per year [24]. No consistent records of late registrations were kept for the period 1983–2003 [24].

We used the numbers of male and female births each year to calculate the annual proportion of male births from 1876 to 2009. Due to the possible inaccuracies in the birth registrations prior to 1961 [21], we ran two separate time-series analyses: one of the full data set from 1876–2009, and a second from 1961–2009. We also calculated the annual proportion of male stillborn babies, defined by Statistics New Zealand as a late foetal loss (the current definition being a birth after the 20th week of gestation or a birth weight of at least 400 g). Stillbirth data from Statistics NZ, including the baby's sex, is derived from birth registration forms. There is some degree of error, since if the sex is not clear or indeterminate the child's sex is classified as ‘male’. However, registrations with indeterminate sex are rare. For example, the rate between April 2009 and March 2011 was 4 per 100,000 births, live and stillbirths combined (personal communication, Anne Howard, Statistical Analyst, Population Statistics Unit, Statistics New Zealand). All were stillbirths with very low weight and early gestation. Annual data on stillbirths were first collected by Statistics New Zealand in 1929, limiting our analyses incorporating the sex ratio of stillbirths to the years 1929–2009. Numbers of stillborn babies ranged from a minimum of 169 in 1973 to a maximum of 971 in 1941. Data on the monthly proportion of male births (but not stillbirths) were available from Statistics New Zealand from 1980 to 2009, which we used for a seasonal analysis.

To obtain a composite measure of temperature in °C for New Zealand, we used data collected by the National Institute of Water and Atmospheric Research (NIWA) at multiple sites from seven locations, three on the North Island (Wellington, Auckland, Masterton) and four from the South Island (Hokitika, Nelson, Lincoln, Dunedin). NIWA is a New Zealand government-owned Crown Research Institute. The seven locations were selected by NIWA as they provide a representative geographical spread across New Zealand and have reliable historical records. The temperature data across these sites were merged by NIWA using a standardized methodology for New Zealand [25], [26] and incorporated into our analyses for the first two hypotheses.

For our analysis on the possible effects of seasonal changes in ambient temperature, six of the seven NIWA sites were used to calculate monthly ambient temperature for New Zealand for the period 1980–2009. The weather station at Hokitika was not included on the recommendation of NIWA, due to the difficulty in constructing a consistent monthly data set for the time period. The average across the six weather stations was calculated in order to generate a composite measure of ambient temperature for each month of the year for the time period 1980–2009. Following Lerchl [18], we also calculated a temperature anomaly series, by subtracting from each observed monthly temperature the relevant monthly average temperature, calculated for each of the 12 months of the year across the 30 years. The temperature anomaly series allows us to investigate the effect of unusually warm or cold months on the proportion of male births, compared to the average for that time of year.

We used transfer function (ARIMA) models [27] to estimate the dynamic effects of temperature and the proportion of male stillbirths on the proportion of males born, following the statistical analyses described by Catalano et al. [11]. In addition, in order to identify any unusual outlying observations or structural changes that are best modelled explicitly as functions of time, we used an intervention analysis approach [28], [29]. Models were tested to ensure a lack of residual autocorrelation using the Ljung-Box portmanteau statistic [30], which indicated that all models discussed below had no residual structure (*p*≥0.234). Computations used SPSS [31].

## Results

**Figure 1:**
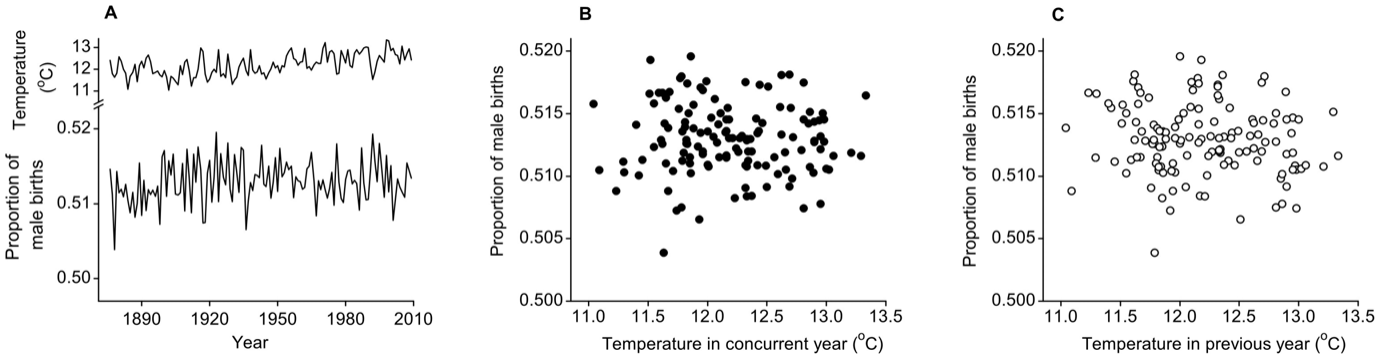
Annual proportion of male births and mean ambient temperature (1876–2009). A. The proportion of male births from 1876–2009 and the mean annual ambient temperature in °C during the same period. The proportion of male births in relation to mean annual ambient temperature is also shown for the B, concurrent and C, previous years.

For our first hypothesis, over the period 1876–2009 the mean annual temperature varied between a low of 11.04°C and a high of 13.33°C. The proportion of males born ranged by 3.17%, from 0.504 in 1878 to 0.520 in 1923 (Fig. 1). We found no significant relationship between the proportion of male births and the mean annual temperature in the concurrent or previous years (*p*≥0.187; Table 1).

**Table 1:** The effects of temperature during the concurrent and previous years on the annual proportion of male births from 1876–2009*.

	*β* (±s.e.)	*t*	*p*
Constant	0.520 (±0.007)	77.02	<0.001
Temperature	1.8×10^−4^ (±0.001)	0.311	0.756
Temperature_ t-1_	0.001 (±0.001)	1.326	0.187

*Selected model required no ARIMA parameters, since there was no residual autocorrelation: Ljung-Box *p* = 0.319. Model *R* ^2^ = 0.015.

The annual proportion of male births in fact shows no temporal structure and behaves like random noise: lag-1 autocorrelation  = −0.037, *p* = 0.667; Ljung-Box statistic  = 17.081 (at 16 lags), *p* = 0.380. Further analysis of the annual proportion of male births is presented in Supporting Text S1, including additional analysis of the residuals from the fitted model, which confirms the model is appropriate. In contrast to the proportion of male births, annual temperature was observed to be strongly positively autocorrelated (lag-1 autocorrelation  = 0.555, *p*<0.001; Ljung-Box statistic  = 181.630 at 16 lags, *p*<0.001).

Errors in reporting annual birth rates may have masked the true proportion of male births occurring in some years between 1876 and 1960 [21]. Thus we repeated our analysis using data on the annual proportion of males born from 1961–2009. Further analysis of the annual proportion of male births for this shorter time series is presented in Supporting Text S2, including additional analysis of the residuals from the fitted model, which confirms the model is appropriate. As with the full data, we found no significant relationship between the proportion of male births and mean annual ambient temperature in the previous year (*p* = 0.362; Table 2A). However, the relationship between the proportion of male births and temperature in the current year was negative and approaching statistical significance (*p* = 0.051; Table 2A).

**Table 2:** The effects of temperature during the concurrent and previous years on the annual proportion of male births from 1961–2009 for: A, all years and B, after removal of three years with high leverage.

A*	*β* (±s.e.)	*t*	*p*
Constant	0.546 (±0.013)	43.34	<0.001
Temperature	−0.002 (±0.001)	−2.008	0.051
Temperature_ t-1_	0.001 (±0.001)	0.921	0.362

B **	*β* (±s.e.)	*t*	*p*
Constant	0.532 (±0.014)	38.96	<0.001
Temperature	−0.001 (±0.001)	−0.634	0.529
Temperature_ t-1_	0.001 (±0.001)	1.104	0.276

*Selected model required no ARIMA parameters, since there was no residual autocorrelation: Ljung-Box *p* = 0.234. Model *R* ^2^ = 0.136.

**Selected model required no ARIMA parameters, since there was no residual autocorrelation: Ljung-Box *p* = 0.953. Model *R* ^2^ = 0.046.

In fact the almost-significant negative relationship is due to observations from only a small number of years during the 1961–2009 period. When examining a scatterplot of the raw data on proportion of male births and ambient temperature in the concurrent year (Supporting Text S3), the years 1976, 1992 and 2001 have high leverage. The lowest and second lowest temperatures in the dataset occurred with the highest and third highest proportion of males born (1992 and 1976, respectively). Further, the lowest proportion of males born occurred with a temperature well above the mean (2001). We therefore ran a second time series analysis in which these three years were removed, the results of which are presented in Table 2B with further analysis presented in Supporting Text S3. Results showed the association between temperature in the concurrent year and the proportion of males born remained negative but clearly non-significant (*p* = 0.529; Table 2B). The association between temperature in the previous year and the proportion of males born remained positive but non-significant (*p* = 0.276; Table 2B).

For the second hypothesis, we re-analysed the subset of data for which we had stillbirth information (1929–2009). We found no significant relationship between the proportion of males born and the mean annual temperature in the concurrent or previous years (*p*≥0.211; Table 3), neither did the proportion of male stillborn babies in the concurrent or previous years have any significant effect on the proportion of male live births (*p*≥0.295; Table 3).

**Table 3:** The effects of temperature and the proportion of male stillbirths during the concurrent and previous years on the annual proportion of male births from 1929–2009*.

	*β* (±s.e.)	*t*	*p*
Constant	0.534 (±0.015)	36.65	<0.001
Temperature	−0.001 (±0.001)	−0.796	0.428
Temperature_ t-1_	0.001 (±0.001)	1.261	0.211
Stillbirths	−0.013 (±0.012)	−1.054	0.295
Stillbirths_ t-1_	−0.008 (±0.012)	−0.707	0.482

*Selected model required no ARIMA parameters, since there was no residual autocorrelation: Ljung-Box *p* = 0.564. Model *R* ^2^ = 0.067.

**Figure 2:**
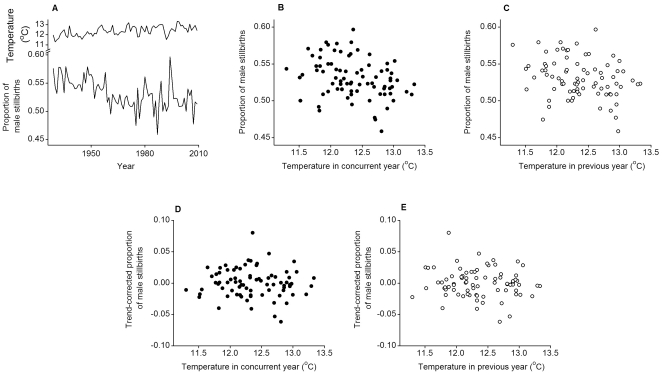
Annual proportion of male stillbirths and mean ambient temperature. A. The proportion of male stillbirths from 1929–2009 and the mean annual ambient temperature in °C during the same period. The proportion of male stillbirths in relation to mean annual ambient temperature is also shown for the B, concurrent and C, previous years. The apparent association in Panels B and C can be explained by the declining trend in the proportion of male stillbirths over time. After removing this temporal trend, there is no association between the residuals from a regression of the proportion of male stillbirths on time, plotted against temperature in the D, concurrent and E, previous years.

We also found no evidence that fewer male stillbirths occurred during warmer concurrent or previous years (*p*≥0.798; Table 4). A declining trend in the proportion of male stillbirths was observed throughout the data (slope  = −5.9×10^−4^±1.3×10^−4^, *p*<0.001; Fig. 2A). A significant positive outlier occurred in 1994 (0.080±0.022, *p*<0.001), which was when the highest proportion of male stillbirths (0.596) was recorded. An apparent association between proportion of male stillbirths and temperature (Figs. 2B, 2C) is completely explained by the declining trend in the proportion of male stillbirths. This conclusion is clear by viewing the residuals from a regression of the proportion of male stillbirths on time, plotted against temperature in concurrent and previous years; those trend-corrected stillbirth proportions show no association with temperature (Figs. 2D, 2E). Further analysis of the annual proportion of male stillbirths is presented in Supporting Text S4, including additional analysis of the residuals from the fitted model, which confirms the model is appropriate.

**Table 4:** The effects of temperature during the concurrent and previous years on the proportion of male stillbirths from 1929–2009*.

	*β* (±s.e.)	*t*	*p*
Constant	0.590 (±0.089)	6.618	<0.001
Temperature	−0.002 (±0.006)	−0.257	0.798
Temperature_ t-1_	0.001 (±0.007)	0.199	0.843

*Outliers/Structural change*			
1994 (Additive)	0.080 (±0.022)	3.706	<0.001
1929 (Local Trend)	−5.9×10^−4^ (±1.3×10^−4^)	−4.414	<0.001

*Selected model required no ARIMA parameters, since there was no residual autocorrelation: Ljung-Box *p* = 0.873. Model *R* ^2^ = 0.387.

For our third hypothesis, following Lerchl [16], [18], we tested whether monthly ambient temperatures, or deviations from those seasonal patterns (i.e. extreme temperatures), were positively related to the monthly proportion of male births over the period 1980–2009. We also tested whether monthly numbers of births, and the monthly proportion of male births, vary in a seasonal manner.

**Figure 3:**
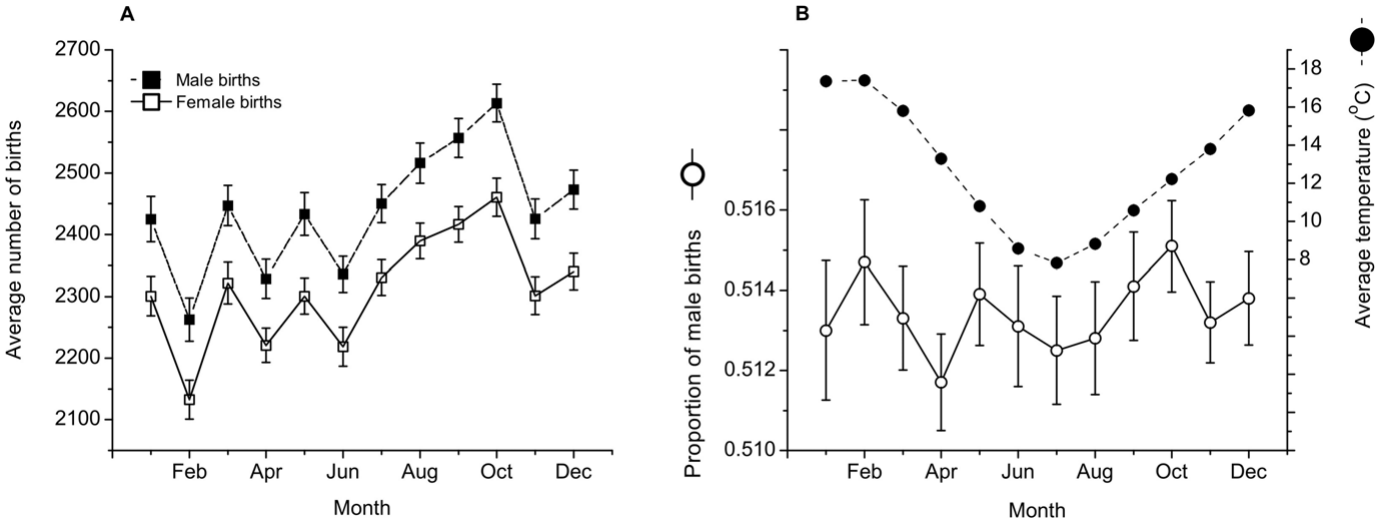
Monthly mean number of births, mean proportion of male births and mean temperature. A. The average numbers of male and female births (±1 standard error) in each month of the year, averaged over 1980–2009 (*n* = 30). B. Average proportion of male births ±1 standard error (left axis) and average temperature ±1 standard error (right axis), averaged over 1980–2009. Temperature is clearly seasonal, while the proportion of male births is not.

**Figure 4:**
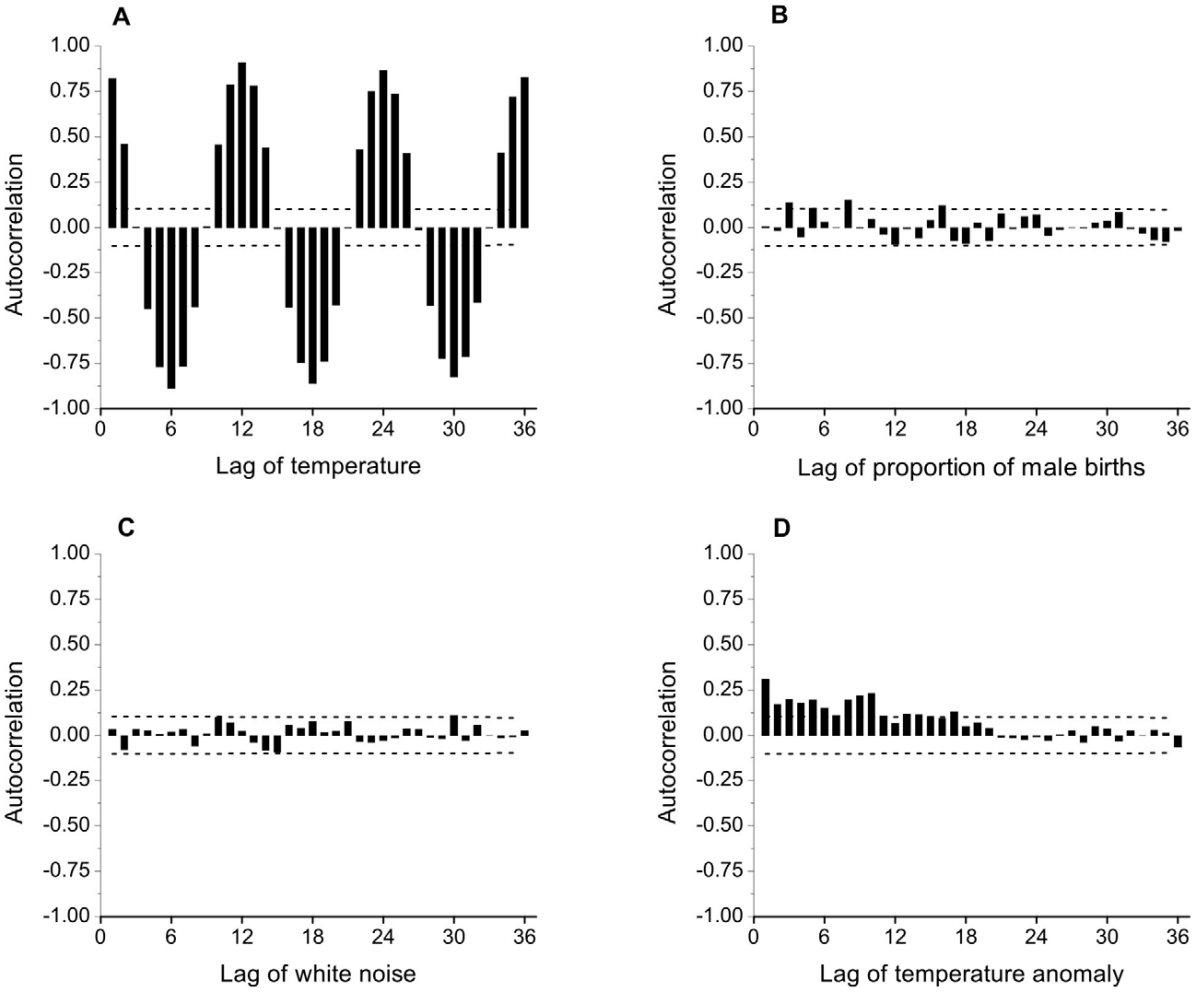
Monthly autocorrelations. Sample autocorrelations, to 36 lags, of: A, monthly NZ temperature (1980–2009); B, monthly NZ proportion of male births (1980–2009); C, a simulated white noise time series of *n* = 360 independent observations; D, monthly NZ temperature anomaly (1980–2009). Dashed lines show 95% confidence interval limits for true correlations of zero; estimates outside these limits are significantly different from zero with *p*<0.05.

As Figure 3A shows, there is a seasonal pattern in the number of births with more males and more females born in the months of August-October than at any other time of year. The patterns of male and female births are very similar throughout the year though, reflected in the lack of any seasonal pattern in the proportion of male births (Fig. 3B). As expected, mean temperature shows a very marked seasonal pattern (Fig. 3B). These general impressions are supported by the autocorrelation functions, to 36 lags, of temperature and proportion of male births (Figs. 4A, 4B). Temperatures 12 months apart (and 24, 36, etc.) are strongly positively correlated, while temperatures 6, 18, 30 months apart are strongly negatively correlated. In contrast lags of the proportion of male births show almost no significant correlations, with only marginal significance at lags 3, 8 and 16. The sizes of the estimated autocorrelations of the proportion of male births are similar to those of a simulated time series of 360 independent, zero-mean Gaussian observations, or white noise (Fig. 4C). As expected following the positive association seen in the annual temperature series, the temperature anomaly series shows modest (significant) positive correlations, extending for approximately 18 lags (Fig. 4D).

**Figure 5:**
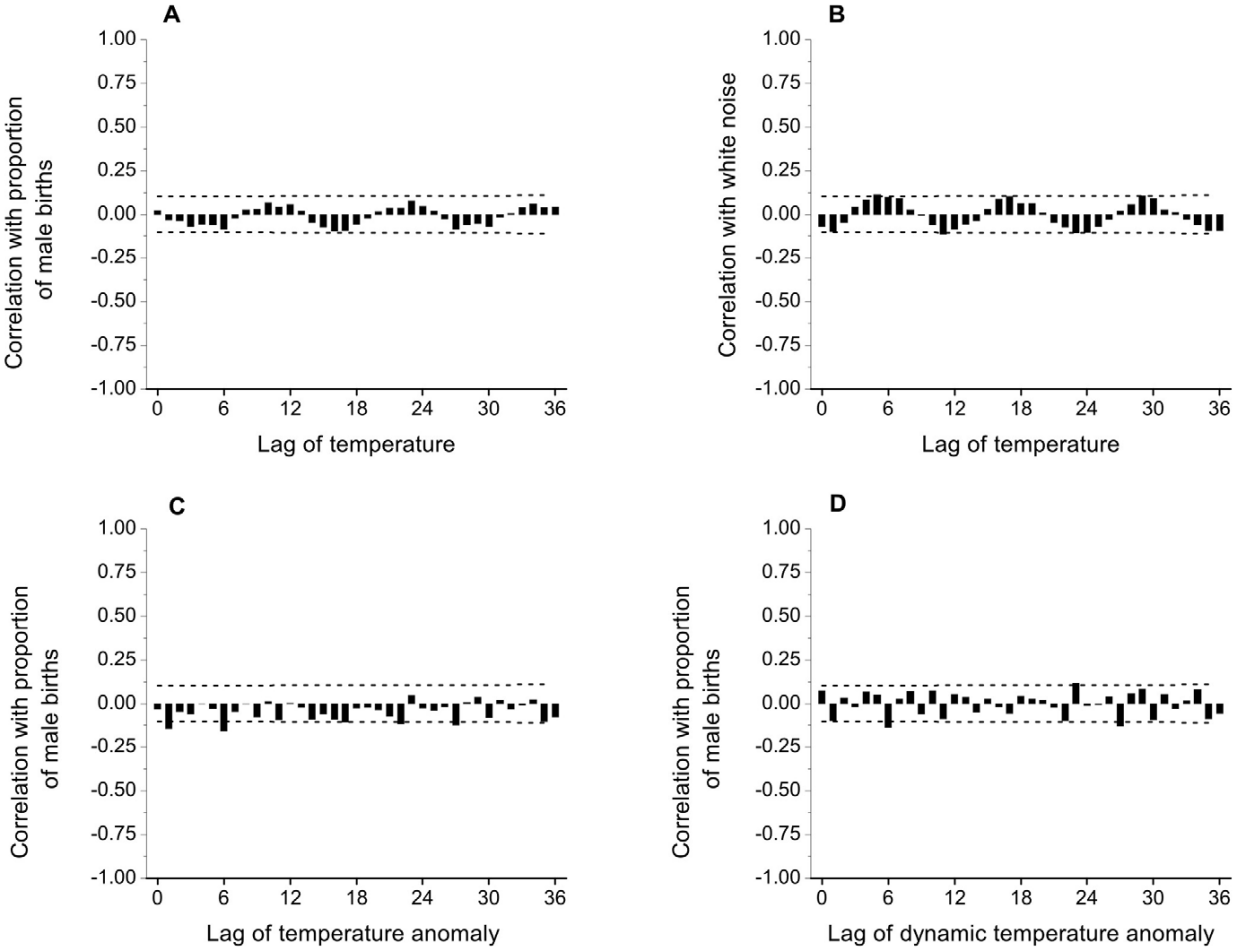
Monthly cross correlations. Sample cross correlations, to 36 lags, of: A, monthly proportion of male births with temperature (1980–2009); B, 360 simulated independent white noise observations with monthly temperature (1980–2009); C, monthly proportion of male births with temperature anomaly (1980–2009); D, monthly proportion of male births with dynamic temperature anomaly (1980–2009). Dashed lines show 95% confidence interval limits for true correlations of zero; estimates outside these limits are significantly different from zero with *p*<0.05.

The cross correlations between the proportion of male births and lags of temperature (to lag 36) are shown in Figure 5A, following the approach of Lerchl [18]. All correlations are small in absolute size, with none statistically significant. There is a cyclical pattern to the estimated cross correlations, yet this is to be expected (e.g. Chapter 12, [27]) because of the very pronounced cyclical pattern in the autocorrelation function of temperature (Fig. 4A). To illustrate this further, the cross correlations between a simulated independent white noise series and lags of temperature (to lag 36) are shown in Figure 5B. These data also display a cyclical pattern, with correlations of similar magnitude to those in Figure 5A.

The cross correlations between the proportion of male births and lags of the temperature anomaly series (to lag 36) are shown in Figure 5C. These cross correlations are almost all not significant, with marginally significant negative correlations only at lags 1, 6, 22 and 27. There are certainly no significant positive correlations at any lag, in contrast to the findings of Lerchl [18]. However, a feature of the correlations presented in Figure 5C is that they are almost all negative, which seems somewhat unexpected. The reason is that the method used to construct the anomaly series, following Lerchl [18], takes an average across all 30 years of data in order to identify departures from ‘trend’. However, time series trends are more appropriately modelled dynamically using decomposition methods [32]–[34], and an alternative temperature anomaly series can be constructed using departures from a dynamic seasonal pattern, following estimation using a centred 12-point moving average (e.g., [34]). The cross correlations between the proportion of male births and lags of the dynamic temperature anomaly series (to lag 36) are shown in Figure 5D. As before, these cross correlations are almost all not significant, with marginally significant correlations only at lags 6, 23 and 27. Now though, similar numbers of positive and negative correlations appear. The overall impression is consistent with that from Figure 5C: there are no significant positive correlations at lags just preceding the time of conception, in contrast to the findings of Lerchl [18]. Thus, neither seasonal variations in temperature nor temperature anomalies (extremes) positively influence the proportion of males born in New Zealand.

## Discussion

This study showed that the impact of concurrent and previous year mean annual ambient temperatures on the proportion of male births in New Zealand (NZ) was not statistically significant from 1876–2009. Changes in the proportion of male births from 1929–2009 were not related to changes in the concurrent or previous year proportion of male stillbirths. In addition, changes in mean annual temperature had no effect on the proportion of male stillbirths. Finally, while we did detect a seasonal pattern in the number of births from 1980 to 2009, the proportion of male births did not display a seasonal pattern and monthly fluctuations in ambient temperature were unrelated to the proportion of male births. Further, monthly temperature anomalies (extremes) did not affect the proportion of male births. Our results therefore clearly do not support the temperature dependent sex allocation hypothesis for people living in NZ.

The results from tests of our first hypothesis differ from other studies in which time-series analyses were used to explore the relationship between the secondary sex ratio (SSR) and fluctuations in mean annual ambient temperature. Studies conducted in Finland and elsewhere in Scandinavia have found that more males are born in warmer years [11], [14] and increases in temperature increased the likelihood of a male foetus surviving [11]. Unlike these Scandinavian studies, fluctuations in mean annual ambient temperature in NZ are unrelated to the rates of male births. NZ's mean annual ambient temperature of 12.195°C over the period 1876–2009 contrasts with the mean annual temperature of 3.352°C observed in Scandinavia from 1865–1914 [11]. However, NZ did exhibit more similarity in the range of annual ambient temperatures (11.04°C–13.33°C, compared to 0.613°C–4.880°C reported for Scandinavia [11]).

New Zealand's demographic data, from the beginning of European settlement, was noted to be of comparable quality to those in the most developed countries of the Western World (p. 10, [20]). However, we note that the annual data for male and female births between 1876 and 1960 are subject to certain problems. For example, there were several years where the reporting of Maori and non-Maori births was inaccurate [21]. While our time series analyses did not detect any statistical outliers in these years, we were concerned we had tested our hypothesis initially on potentially inaccurate data. Consequently, we tested for effects of mean annual ambient temperature on the proportion of males born from 1961–2009. Results for the shorter time series revealed no significant relationship between the proportion of males born and the previous year temperature. However, there was a marginally significant negative relationship between the ambient temperature in the concurrent year and the proportion of males born (*p* = 0.051). This result has emerged due to the effect of just a few years within this shorter time series. Thus, when we ran the time-series analyses for a second time after removing just three years of high-leverage data, while the negative relationship remained the *p*-value changed from 0.051 to 0.529. Therefore, if anything, these findings are the opposite from those reported in Scandinavia and may reflect differences between hemispheres in the seasonality of reproduction, as has been previously documented [35]–[37].

Our study incorporated a similar geographic range and range of fluctuations in temperature as the studies which have found a significant relationship between annual SSR and mean temperature [7], [11], [14]. Nevertheless, our use of a composite measure of annual ambient temperature may hide or mask any possible seasonal effects of climate on sex allocation. Lerchl [16] analysed the SSR in Germany from 1946–1995 on a monthly basis and found a seasonal pattern in the SSR, so that the male bias increased in April to June, suggesting that conception of boys increased during summer months (July to September). Following this analysis, Lerchl [18] tested whether ambient temperature was linked with the seasonal SSR and identified that the temperature 10 and 11 months prior to birth was significantly positively correlated with the SSR. We did find a seasonal pattern in birth rates, with more births occurring in August to October, suggesting more conceptions occur in the summer in NZ (December–February). However, there was no seasonal variation in the proportion of male births and no relationship between the proportion of male births and the highly seasonal temperature variations, which showed clear seasonal correlations. In fact there was very little structure in the monthly SSR, so that it was similar to a simulated series of independent white noise. Therefore, as in our time-series analyses of annual proportions of male births and stillbirths, we found no support for the hypothesis that seasonal variation in ambient temperature at the time of conception is related to the proportion of male births in New Zealand.

While our findings do not support those from other studies testing for patterns within countries, they are consistent with results from cross-sectional studies testing for associations between temperature and the SSR conducted on a broader global scale. Navara [13] used global data on the SSR from 202 countries from 1997–2006 and found that the SSR was significantly less male-biased at tropical latitudes than at temperate and subarctic latitudes. In African countries and among women of sub-Saharan African descent, it has been shown that the SSR is less male biased than among non-African women [38], [39]. In warmer climates in Europe more males were born; however, this trend was not observed in North American cultures [40]. Our results for NZ follow the general global pattern of male-bias in the proportion of male births that has been observed in other temperate climates [13].

It could be argued that with the advent of better housing and central heating in modern homes, ambient temperature may have exerted a different effect on population health in the late 1800 s than in more recent years. However, in NZ central heating is still uncommon. In a survey conducted in 2004 of 397 randomly selected houses from throughout NZ, only 5% had central heating [41]. Thus, we think that our failure to find a positive effect of mean annual ambient temperature on the proportion of male births is not an artefact of modern housing in NZ. There are other documented influences of temperature on fertility and the SSR, such as occupational exposure of the testes to extreme temperatures (e.g. bakers) [12], [42]. It may be fruitful to investigate these potential influences in future research. Further, there is no published research that investigates the effect of unusually hot temperatures on SSR in hot climates, in contrast to the known positive relationship between high ambient temperature and mortality [8].

We did detect a decline in the proportion of male stillbirths from 1929–2009. However, time-series analyses did not reveal any relationship between the proportion of male stillbirths and mean annual ambient temperature. This is in contrast to a previous study comparing data from Denmark, Finland, Norway and Sweden, where increases in ambient temperature significantly increased the SSR but reduced the life-span of the male cohort at one year of age, suggesting that fluctuations in ambient temperature can induce stress during gestation, resulting in the loss of male foetuses [11]. It is important to note that changes in definitions of a stillbirth from 1995 onwards have slightly increased the number of stillbirths that have been logged in NZ, although overall numbers are still low (e.g., 384 total stillbirths in 2009). Prior to 1995, a stillbirth was only logged if the miscarriage occurred after 28 weeks. From 1995 onwards, the definition of a stillbirth was changed to include all miscarriages from 20 weeks of gestation onwards or a foetal body weight of at least 400 g. A further issue concerns those stillbirths where the sex was indeterminate, though Statistics New Zealand suggests such indeterminate births occur in only 4 per 100,000 total births. Thus, it seems likely that indeterminate births had little influence on our analyses and conclusions. The decline in stillbirths from 1929 to 2009 may reflect improvements in pre-natal healthcare in NZ that helped reduce gestational stresses. Indeed, in the 1920 s major changes in maternity and antenatal care were instigated in NZ to address the high rate of maternal deaths around childbirth. Antenatal clinics were formed and new regulations introduced to control hygiene and delivery services in maternity hospitals [22], which significantly lowered maternal perinatal mortality. For example, the perinatal mortality rate among Maori halved between 1946 and 1966 (p. 247, [43]). Other improvements, such as the introduction of free immunization against rubella from 1970 onwards for women of child bearing age, may also have contributed to a reduction in stillbirths.

In conclusion, we did not find support for the hypothesis that there is a positive relationship between ambient temperatures in the concurrent or previous year and the proportion of males born. We also did not find support for the hypothesis that foetal loss is negatively associated with ambient temperature. Where we did find weak relationships in the data, such as between the current year's temperature and the proportion of males born from 1961 to 2009, the association was negative.

## Supporting Information

Text S1 This file contains the sample autocorrelation function (ACF) and sample partial autocorrelation function (PACF) for the proportion of males born in New Zealand from 1876-2009. There is no significant temporal structure. Also included are the ACF and PACF of the residuals from the transfer function (ARIMA) model used to estimate the effects of temperature on the proportion of males born. The lack of residual structure confirms the model is appropriate for the data.
(DOC)

Text S2 This file contains the sample autocorrelation function (ACF) for the proportion of males born in New Zealand from 1961-2009. The lags at 9 and 11 years are statistically significant. However, there is no significant temporal structure at lags with any biological relevance to the hypotheses under examination. Also included are the ACF and PACF of the residuals from the transfer function (ARIMA) model used to estimate the effects of temperature on the proportion of males born using this shorter time series. The lack of residual structure confirms the model is appropriate for the data.
(DOC)

Text S3 This file contains further analysis of the proportion of males born from 1961-2009. A scatterplot shows there are three years with high leverage (1976, 1992, 2001). After removing these data we re-ran the time series analysis. The sample autocorrelation function (ACF) and sample partial autocorrelation function (PACF) show that now there is no significant temporal structure. Also, the ACF and PACF of the residuals from the transfer function (ARIMA) model used to estimate the effects of temperature on the proportion of males born are shown. The lack of residual structure confirms the model is appropriate for the data.
(DOC)

Text S4 This file contains the sample autocorrelation function (ACF) and sample partial autocorrelation function (PACF) for the proportion of New Zealand male stillbirths from 1929-2009. There are clear positive autocorrelations, due to the downward trend in the proportion of stillbirths. After detrending the proportions using a simple regression on year, the ACF shows no correlations are significantly different from zero. The ACF and PACF of the residuals from the transfer function (ARIMA) model used to estimate the effects of temperature on the proportion of male stillbirths are also shown. The lack of residual structure confirms all positive correlations in the proportion of male stillbirths have been successfully modeled.
(DOC)
