# Supplementary material for: Whatever the Weather: Ambient Temperature Does Not Influence the Proportion of Males Born in New Zealand
Source: PLoS One. 2011 Sep 21;6(9):e25064. doi: 10.1371/journal.pone.0025064 (PMC3177861; doi:10.1371/journal.pone.0025064)
Supplement: Text S1 — This file contains the sample autocorrelation function (ACF) and sample partial autocorrelation function (PACF) for the proportion of males born in New Zealand from 1876-2009. There is no significant temporal structure. Also included are the ACF and PACF of the residuals from the transfer function (ARIMA) model used to estimate the effects of temperature on the proportion of males born. The lack of residual structure confirms the model is appropriate for the data. (DOC) [file pone.0025064.s001.doc]

**Supporting Text S1**.

*Sample autocorrelation function (ACF) for proportion of NZ male births (labelled ssr), 1876-2009. There is no significant temporal structure.*

| **Autocorrelations** | | | | | |
| --- | --- | --- | --- | --- | --- |
| Series:ssr | | | | | |
| Lag | Autocorrelation | Std. Error^a^ | Box-Ljung Statistic | | |
|  |  |  | Value | df | Sig.^b^ |
| 1 | -.037 | .085 | .186 | 1 | .667 |
| 2 | -.052 | .085 | .556 | 2 | .757 |
| 3 | .169 | .085 | 4.536 | 3 | .209 |
| 4 | -.020 | .084 | 4.590 | 4 | .332 |
| 5 | .070 | .084 | 5.287 | 5 | .382 |
| 6 | .038 | .084 | 5.495 | 6 | .482 |
| 7 | -.038 | .083 | 5.699 | 7 | .575 |
| 8 | .080 | .083 | 6.620 | 8 | .578 |
| 9 | .034 | .083 | 6.787 | 9 | .659 |
| 10 | -.026 | .082 | 6.889 | 10 | .736 |
| 11 | .158 | .082 | 10.582 | 11 | .479 |
| 12 | .134 | .082 | 13.278 | 12 | .349 |
| 13 | -.035 | .081 | 13.463 | 13 | .413 |
| 14 | -.121 | .081 | 15.672 | 14 | .334 |
| 15 | .094 | .081 | 17.039 | 15 | .317 |
| 16 | -.017 | .080 | 17.081 | 16 | .380 |
| a. The underlying process assumed is independence (white noise). | | | | | |
| b. Based on the asymptotic chi-square approximation. | | | | | |

*Sample partial autocorrelation function (PACF) for proportion of NZ male births (labelled ssr), 1876-2009. Consistent with the ACF, there is no significant temporal structure.*

| **Partial Autocorrelations** | | |
| --- | --- | --- |
| Series:ssr | | |
| Lag | Partial Autocorrelation | Std. Error |
| 1 | -.037 | .086 |
| 2 | -.053 | .086 |
| 3 | .166 | .086 |
| 4 | -.011 | .086 |
| 5 | .089 | .086 |
| 6 | .014 | .086 |
| 7 | -.023 | .086 |
| 8 | .056 | .086 |
| 9 | .029 | .086 |
| 10 | -.013 | .086 |
| 11 | .139 | .086 |
| 12 | .144 | .086 |
| 13 | -.011 | .086 |
| 14 | -.175 | .086 |
| 15 | .049 | .086 |
| 16 | -.041 | .086 |

*ACF and PACF for residuals from the model for proportion of NZ male births (as summarised in Table 1, Ljung-Box p = 0.319). The lack of residual structure confirms the model is appropriate for the data.*
