# Supplementary material for: Whatever the Weather: Ambient Temperature Does Not Influence the Proportion of Males Born in New Zealand
Source: PLoS One. 2011 Sep 21;6(9):e25064. doi: 10.1371/journal.pone.0025064 (PMC3177861; doi:10.1371/journal.pone.0025064)
Supplement: Text S2 — This file contains the sample autocorrelation function (ACF) for the proportion of males born in New Zealand from 1961-2009. The lags at 9 and 11 years are statistically significant. However, there is no significant temporal structure at lags with any biological relevance to the hypotheses under examination. Also included are the ACF and PACF of the residuals from the transfer function (ARIMA) model used to estimate the effects of temperature on the proportion of males born using this shorter time series. The lack of residual structure confirms the model is appropriate for the data. (DOC) [file pone.0025064.s002.doc]

**Supporting Text S2**.

*Sample autocorrelation function (ACF) for proportion of NZ male births (labelled ssr), 1961-2009.*

*The lags at 9 and 11 years are statistically significant. However, there is no significant temporal structure at lags with any biological relevance to the hypotheses under examination.*

| **Autocorrelations** | | | | | |
| --- | --- | --- | --- | --- | --- |
| Series:ssr | | | | | |
| Lag | Autocorrelation | Std. Error^a^ | Box-Ljung Statistic | | |
|  |  |  | Value | df | Sig.^b^ |
| 1 | .018 | .139 | .017 | 1 | .897 |
| 2 | -.092 | .137 | .471 | 2 | .790 |
| 3 | -.047 | .136 | .592 | 3 | .898 |
| 4 | .020 | .134 | .613 | 4 | .962 |
| 5 | .112 | .133 | 1.321 | 5 | .933 |
| 6 | -.134 | .131 | 2.360 | 6 | .884 |
| 7 | -.109 | .130 | 3.064 | 7 | .879 |
| 8 | .029 | .128 | 3.117 | 8 | .927 |
| 9 | -.298 | .127 | 8.666 | 9 | .469 |
| 10 | -.205 | .125 | 11.364 | 10 | .330 |
| 11 | .346 | .123 | 19.225 | 11 | .057 |
| 12 | .020 | .122 | 19.251 | 12 | .083 |
| 13 | -.019 | .120 | 19.276 | 13 | .115 |
| 14 | -.235 | .118 | 23.229 | 14 | .057 |
| 15 | .021 | .117 | 23.262 | 15 | .079 |
| 16 | .163 | .115 | 25.267 | 16 | .065 |
| a. The underlying process assumed is independence (white noise). | | | | | |
| b. Based on the asymptotic chi-square approximation. | | | | | |

*ACF and PACF for residuals from the model for proportion of NZ male births (as summarised in Table 2A in the main text). The lack of residual structure confirms the model is appropriate for the data.*
