# Supplementary material for: Whatever the Weather: Ambient Temperature Does Not Influence the Proportion of Males Born in New Zealand
Source: PLoS One. 2011 Sep 21;6(9):e25064. doi: 10.1371/journal.pone.0025064 (PMC3177861; doi:10.1371/journal.pone.0025064)
Supplement: Text S3 — This file contains further analysis of the proportion of males born from 1961-2009. A scatterplot shows there are three years with high leverage (1976, 1992, 2001). After removing these data we re-ran the time series analysis. The sample autocorrelation function (ACF) and sample partial autocorrelation function (PACF) show that now there is no significant temporal structure. Also, the ACF and PACF of the residuals from the transfer function (ARIMA) model used to estimate the effects of temperature on the proportion of males born are shown. The lack of residual structure confirms the model is appropriate for the data. (DOC) [file pone.0025064.s003.doc]

**Supporting Text S3**.

*The marginal significance (p = 0.051) of the relationship between the proportion of male births (labelled ssr) and temperature (labelled temp) is due to observations from only a small number of years during the 1961-2009 period. We provide two scatterplots below. The upper panel shows the full data set, from which it is clear the years 1976, 1992 and 2001 have high leverage. The lowest and second lowest temperatures in the dataset occurred with the highest and third highest proportion of males born (1992 and 1976, respectively). The lowest proportion of males born occurred with a temperature well above the mean (2001). The lower panel shows the revised data set excluding the three years of data with high leverage.*

*After removing data from the three years with high leverage, we re-ran the time series analysis again. The results are summarised in the main body of the text and in Table 2B. Further results are given below. It is clear those three years of data are highly influential.*

*Sample autocorrelation function (ACF) for proportion of NZ male births (labelled ssr), 1961-2009, excluding 1976, 1992 and 2001.*

| **Autocorrelations** | | | | | |
| --- | --- | --- | --- | --- | --- |
| Series:ssr | | | | | |
| Lag | Autocorrelation | Std. Error^a^ | Box-Ljung Statistic | | |
|  |  |  | Value | df | Sig.^b^ |
| 1 | -.034 | .143 | .056 | 1 | .813 |
| 2 | -.034 | .141 | .113 | 2 | .945 |
| 3 | -.029 | .140 | .157 | 3 | .984 |
| 4 | -.067 | .138 | .393 | 4 | .983 |
| 5 | .041 | .136 | .482 | 5 | .993 |
| 6 | -.008 | .135 | .486 | 6 | .998 |
| 7 | .070 | .133 | .764 | 7 | .998 |
| 8 | -.119 | .131 | 1.586 | 8 | .991 |
| 9 | -.207 | .129 | 4.137 | 9 | .902 |
| 10 | .186 | .128 | 6.263 | 10 | .793 |
| 11 | .005 | .126 | 6.264 | 11 | .855 |
| 12 | .084 | .124 | 6.719 | 12 | .876 |
| 13 | -.080 | .122 | 7.147 | 13 | .894 |
| 14 | .026 | .120 | 7.194 | 14 | .927 |
| 15 | -.087 | .118 | 7.738 | 15 | .934 |
| 16 | -.032 | .117 | 7.812 | 16 | .954 |
| a. The underlying process assumed is independence (white noise). | | | | | |
| b. Based on the asymptotic chi-square approximation. | | | | | |


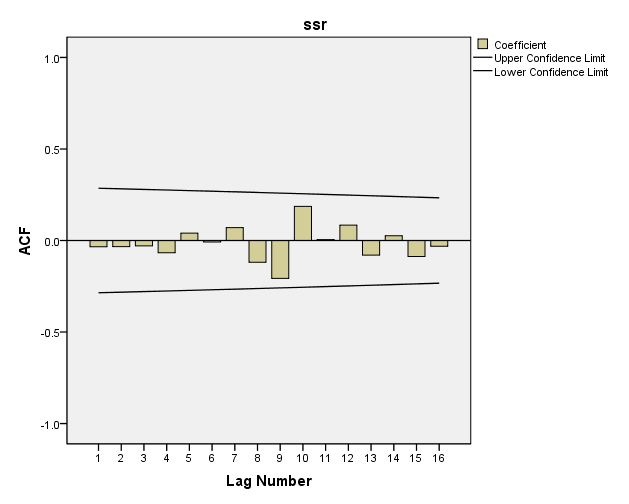


*Sample partial autocorrelation function (PACF) for proportion of NZ male births (labelled ssr), 1961-2009, excluding 1976, 1992 and 2001. Consistent with the ACF, there is no significant temporal structure.*

| **Partial Autocorrelations** | | |
| --- | --- | --- |
| Series:ssr | | |
| Lag | Partial Autocorrelation | Std. Error |
| 1 | -.034 | .147 |
| 2 | -.035 | .147 |
| 3 | -.032 | .147 |
| 4 | -.071 | .147 |
| 5 | .034 | .147 |
| 6 | -.012 | .147 |
| 7 | .068 | .147 |
| 8 | -.119 | .147 |
| 9 | -.210 | .147 |
| 10 | .174 | .147 |
| 11 | .007 | .147 |
| 12 | .065 | .147 |
| 13 | -.095 | .147 |
| 14 | .061 | .147 |
| 15 | -.092 | .147 |
| 16 | -.010 | .147 |

*
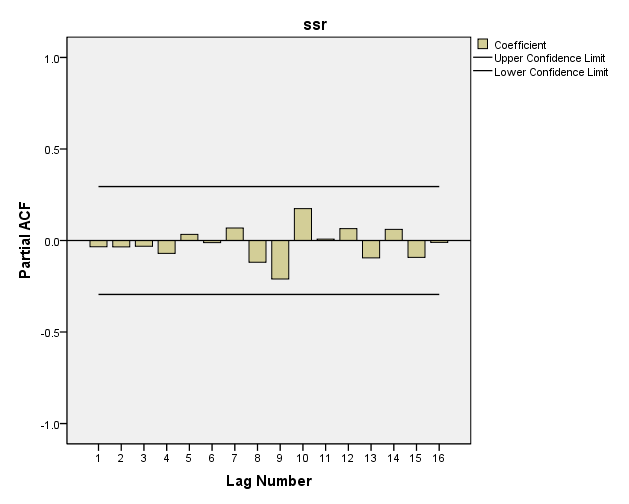
*

*ACF and PACF for residuals from the model for proportion of NZ male births, excluding 1976, 1992 and 2001 (as summarised in Table 2B in the text). The lack of residual structure confirms the model is appropriate for the data (Ljung-Box p = 0.953).*
