# Supplementary material for: Whatever the Weather: Ambient Temperature Does Not Influence the Proportion of Males Born in New Zealand
Source: PLoS One. 2011 Sep 21;6(9):e25064. doi: 10.1371/journal.pone.0025064 (PMC3177861; doi:10.1371/journal.pone.0025064)
Supplement: Text S4 — This file contains the sample autocorrelation function (ACF) and sample partial autocorrelation function (PACF) for the proportion of New Zealand male stillbirths from 1929-2009. There are clear positive autocorrelations, due to the downward trend in the proportion of stillbirths. After detrending the proportions using a simple regression on year, the ACF shows no correlations are significantly different from zero. The ACF and PACF of the residuals from the transfer function (ARIMA) model used to estimate the effects of temperature on the proportion of male stillbirths are also shown. The lack of residual structure confirms all positive correlations in the proportion of male stillbirths have been successfully modeled. (DOC) [file pone.0025064.s004.doc]

**Supporting Text S4**.

*Sample autocorrelation function (ACF) for annual proportion of NZ male stillbirths (labelled stillbirth_ssr), 1929-2009. There are clear positive autocorrelations due to the downward trend visible in Fig. 2A and reported in Table 4.*

| **Autocorrelations** | | | | | |
| --- | --- | --- | --- | --- | --- |
| Series:stillbirth_ssr | | | | | |
| Lag | Autocorrelation | Std. Error^a^ | Box-Ljung Statistic | | |
|  |  |  | Value | df | Sig.^b^ |
| 1 | .394 | .109 | 13.046 | 1 | .000 |
| 2 | .363 | .108 | 24.285 | 2 | .000 |
| 3 | .291 | .108 | 31.598 | 3 | .000 |
| 4 | .313 | .107 | 40.165 | 4 | .000 |
| 5 | .262 | .106 | 46.227 | 5 | .000 |
| 6 | .224 | .106 | 50.719 | 6 | .000 |
| 7 | .171 | .105 | 53.388 | 7 | .000 |
| 8 | .236 | .104 | 58.500 | 8 | .000 |
| 9 | .166 | .103 | 61.074 | 9 | .000 |
| 10 | .304 | .103 | 69.810 | 10 | .000 |
| 11 | .190 | .102 | 73.285 | 11 | .000 |
| 12 | .226 | .101 | 78.243 | 12 | .000 |
| 13 | .145 | .101 | 80.330 | 13 | .000 |
| 14 | .255 | .100 | 86.830 | 14 | .000 |
| 15 | .207 | .099 | 91.212 | 15 | .000 |
| 16 | .210 | .098 | 95.755 | 16 | .000 |
| a. The underlying process assumed is independence (white noise). | | | | | |
| b. Based on the asymptotic chi-square approximation. | | | | | |

*Sample partial autocorrelation function (PACF) for proportion of NZ male stillbirths (labelled stillbirth_ssr), 1929-2009.*

| **Partial Autocorrelations** | | |
| --- | --- | --- |
| Series:stillbirth_ssr | | |
| Lag | Partial Autocorrelation | Std. Error |
| 1 | .394 | .111 |
| 2 | .246 | .111 |
| 3 | .109 | .111 |
| 4 | .145 | .111 |
| 5 | .060 | .111 |
| 6 | .020 | .111 |
| 7 | -.016 | .111 |
| 8 | .104 | .111 |
| 9 | -.010 | .111 |
| 10 | .196 | .111 |
| 11 | -.016 | .111 |
| 12 | .042 | .111 |
| 13 | -.050 | .111 |
| 14 | .118 | .111 |
| 15 | .027 | .111 |
| 16 | .026 | .111 |

*ACF for annual proportion of NZ male stillbirths, 1929-2009,* *after detrending using a simple regression on year. No correlations are significantly different from zero. The final model for proportion of male stillbirths also includes possible effects of temperature and lagged temperature; see summary of Table 4 residuals below.*

| **Autocorrelations** | | | | | |
| --- | --- | --- | --- | --- | --- |
| Series:Unstandardized Residual | | | | | |
| Lag | Autocorrelation | Std. Error^a^ | Box-Ljung Statistic | | |
|  |  |  | Value | df | Sig.^b^ |
| 1 | .181 | .109 | 2.744 | 1 | .098 |
| 2 | .141 | .108 | 4.432 | 2 | .109 |
| 3 | .061 | .108 | 4.749 | 3 | .191 |
| 4 | .104 | .107 | 5.685 | 4 | .224 |
| 5 | .051 | .106 | 5.913 | 5 | .315 |
| 6 | .001 | .106 | 5.914 | 6 | .433 |
| 7 | -.062 | .105 | 6.264 | 7 | .509 |
| 8 | .051 | .104 | 6.507 | 8 | .591 |
| 9 | -.023 | .103 | 6.556 | 9 | .683 |
| 10 | .186 | .103 | 9.828 | 10 | .456 |
| 11 | .046 | .102 | 10.029 | 11 | .528 |
| 12 | .104 | .101 | 11.075 | 12 | .523 |
| 13 | -.004 | .101 | 11.077 | 13 | .604 |
| 14 | .153 | .100 | 13.433 | 14 | .493 |
| 15 | .073 | .099 | 13.970 | 15 | .528 |
| 16 | .040 | .098 | 14.133 | 16 | .589 |
| a. The underlying process assumed is independence (white noise). | | | | | |
| b. Based on the asymptotic chi-square approximation. | | | | | |

*ACF and PACF for residuals from the model for proportion of NZ male stillbirths, summarised in Table 4, showing all positive correlations in the proportion of male stillbirths have been successfully modelled (Ljung-Box p = 0.873).*
